# Influences of Seasonal Influenza Disease Perceptions, Altruism, Family Harmony, and Information Exposure on Social Media on Behavioral Intention to Receive Seasonal Influenza Vaccination Among Parents in China: Findings of a Population-Based Survey

**DOI:** 10.3390/vaccines14010013

**Published:** 2025-12-22

**Authors:** Hongbiao Chen, Liwen Ding, Lixian Su, Minjie Zhang, Yadi Lin, Yuan Fang, Weijun Peng, He Cao, Zixin Wang

**Affiliations:** 1 Longhua District Centre for Disease Control and Prevention, Shenzhen 518055, China; 2 Centre for Health Behaviours Research, JC School of Public Health and Primary Care, The Chinese University of Hong Kong, Hong Kong, China; 3 Futian District Maternal and Child Health Hospital, Shenzhen 518034, China; 4 Department of Health and Physical Education, The Education University of Hong Kong, Hong Kong, China

**Keywords:** seasonal influenza vaccination, behavioral intention, illness representation, altruism, family harmony, social media, Socioecological Model, parents, China

## Abstract

Background: Promoting seasonal influenza vaccination among parents may help increase the coverage of seasonal influenza vaccination among both parents and children. This study aims to investigate determinants of behavioral intention to receive a seasonal influenza vaccination among parents of children aged 0–15 years to protect themselves. Methods: A cross-sectional survey was conducted among parents of children aged 0 to 15 years with administrative health records in Shenzhen, China, between September and October 2024. Participants were recruited through multistage random sampling. First, 10 community health centers were randomly selected in Shenzhen. Within each selected center, 200 parents were randomly selected. Multivariate logistic regression models were fitted. Results: Among 1504 parents, 47.6% intended to receive a seasonal influenza vaccination in the next year. After adjusting for significant background characteristics, parents’ intention to receive a seasonal influenza vaccination was associated with a higher intention to vaccinate their children against seasonal influenza (AOR: 20.39). At the individual level, eight items measuring illness representations of seasonal influenza were associated with higher odds of intending to receive such a vaccine (AOR: 1.15–1.25), including identity (identifying symptoms), timeline, negative consequences, personal and treatment control, concern, negative emotions, and coherence. At the interpersonal level, parents who had higher levels of general and family-oriented altruism (AOR: 1.10–2.47), better family harmony (AOR: 1.07), higher exposure to information related to seasonal influenza on social media (AOR: 1.24–1.38), and thoughtful consideration of information veracity (AOR: 1.33) were more likely to report an intention. Conclusions: There are strong needs to promote seasonal influenza vaccination among parents in China.

## 1. Introduction

Worldwide, there are approximately 1 billion people infected with seasonal influenza every year, with 3–5 million severe cases and 290,000–650,000 respiratory deaths [1]. In China, approximately 3.5 million cases of influenza-like illness are reported annually, with around 80,000 influenza-associated excess respiratory deaths [2,3]. The highest prevalence of Influenza-like illness was observed among adults aged 25–59 years, followed by children aged 0–4 and 5–14 years, accounting for 22%, 14%, and 10% of the total number of Influenza-like illness cases during the peak seasons [2]. Influenza-like illness results in a substantial burden on healthcare systems and a high medical cost [4].

Seasonal influenza vaccination is an important measure for preventing seasonal influenza and reducing all-cause mortality that has been proven to be effective, safe, and well-tolerated for all age groups [5,6]. Two types of vaccines—trivalent and quadrivalent vaccines—are available globally. Over 60% of countries have incorporated seasonal influenza vaccination into their national immunization programs [7]. In China, residents aged 6 months and older are recommended to receive a seasonal influenza vaccination annually [8]. However, such a vaccine is not yet included in the National Immunization Program in China [8]. Some Chinese cities (e.g., Shenzhen) provide free seasonal influenza vaccines to primary and secondary school students, residents aged 60 years or above, and healthcare workers [9]. Other residents can receive a self-financed trivalent or quadrivalent inactivated influenza vaccine (IIV3 or IIV4) or trivalent live attenuated influenza vaccine (LAIV3) at a cost of CNY 45 (USD 6.2) to CNY 153 (USD 22) per dose [8]. The cost of seasonal influenza vaccination corresponds to 1.1–3.8% of the average cost of living per month for one person in Shenzhen (around CNY 4000 or USD 556). However, seasonal influenza vaccination coverage remains low in China; the lowest coverage was observed in adults aged 18–59 years (6.7–12.7%) and children aged under 18 years (25.1–35.7%) [10,11].

Parents have an important influence on their children’s seasonal influenza vaccination uptake, as they are the main decision makers for children’s vaccination [12]. Previous studies showed that parents who have received a seasonal influenza vaccination are more likely to vaccinate their children against seasonal influenza [13,14,15]. However, only 24.8–36.7% of parents in China have received a seasonal influenza vaccination, while 34.1–43.2% of them are hesitant to vaccinate their children against seasonal influenza [16,17]. Therefore, promoting seasonal influenza vaccination among parents may be an effective measure to increase seasonal influenza vaccination coverage among both parents and children, thereby achieving two goals with one strike. Determinants of seasonal influenza vaccination uptake among adults in general and children under 18 years in China have been studied [16,18,19,20]. However, relatively few studies have examined the facilitators of and barriers to parents receiving a seasonal influenza vaccination to protect themselves. Determinants reported by these studies are limited to socio-demographic characteristics (e.g., parents’ and children’s age, household income, and number of children), vaccination history (e.g., COVID-19 vaccination and seasonal influenza vaccination uptake in the last flu season), and parents’ knowledge and perception of seasonal influenza vaccination (e.g., perceived vaccine efficacy, knowledge of priority groups and timing for such vaccinations, and concerns about safety) [21,22].

This study examined the determinants of behavioral intention to receive a seasonal influenza vaccination among parents at both the individual and interpersonal levels under the Socio-ecological Model [23]. This model is commonly used to understand determinants of health-related behaviors, including vaccination behaviors [24,25,26]. This study considered illness representation of seasonal influenza at the individual level. Illness representation refers to how individuals look at a disease or condition, which has been widely used to explain health behavior [27]. The Common-sense Model of Self-regulation suggests that cognitive and emotional representation are activated at the same time when people face a health threat. The cognitive representation is used to regulate the objective threat, and the emotional representation regulates emotions [28]. Both types of illness representations lead to strategies to cope with health threats. Previous studies have found illness representation to be a determinant of the uptake of various types of vaccination, including seasonal influenza vaccination uptake among the general population and older adults [29,30]. It is possible that parents consider receiving a seasonal influenza vaccination for themselves as a coping strategy to regulate cognitive and/or emotional representations of seasonal influenza. At the interpersonal level, we considered altruism, family harmony, and information exposure through social media platforms. Altruism is defined as a desire to benefit someone other than oneself [31]. The triggering of altruism and altruistic behaviors is a useful approach to promote vaccination uptake [32]. Since most Chinese parents live with their children and older adults, achieving herd immunity through vaccination could indirectly protect household members. Observational studies have demonstrated that altruism is associated with parents’ intention to vaccinate their children against influenza [33,34]. A randomized controlled trial showed that altruism-eliciting intervention was more effective than the control condition in increasing intention to receive a COVID-19 vaccination among younger adults [35]. Family harmony is one of the core values in traditional Chinese culture. It emphasizes harmonious, cooperative, and supportive family relationships. Individuals living in a harmonious family are more likely to adopt healthy behaviors (e.g., healthy diet, active lifestyle, and the use of mobile and wireless technologies to support health services) [36,37,38] and to refrain from risky behaviors (e.g., smoking, alcohol consumption, and excessive screen time) [36,39,40,41]. One study explored the relationship between family harmony and human papillomavirus vaccine initiation among adult women but found no significant association [42]. No studies have yet investigated the association between family harmony and seasonal influenza vaccination uptake among parents. Social media platforms serve as a significant source for health-related information, with trending topics including seasonal influenza outbreaks [43]. A study in China found that individuals with a higher frequency of exposure to negative content about vaccinations on social media were less likely to receive a seasonal influenza vaccination [44]. Previous studies have shown that a higher frequency of thoughtful consideration could mitigate the negative impact of misinformation on vaccination uptake, in addition to being associated with increased intention to receive a COVID-19 vaccination.

The aim of this study was to examine the behavioral intention to receive seasonal influenza vaccination among parents with at least one child under 15 years of age in Shenzhen, China. This study also investigated potential determinants of parents’ intention to receive a seasonal influenza vaccination at both individual (illness representations) and interpersonal levels (altruism, family harmony, information exposure on social media, and thoughtful consideration of the veracity of information). It is hypothesized that these factors are significantly associated with parents’ intention to receive a seasonal influenza vaccination for themselves.

## 2. Methods

### 2.1. Study Design

This is a cross-sectional survey among parents living in Shenzhen conducted between September and October 2024. Shenzhen is a special economic zone and one of the most developed cities in mainland China. It had a population of 17 million in 2021.

### 2.2. Participants and Data Collection

The inclusion criteria for this study were adults (1) aged 18 years old or above (2) with least one child aged 0–15 years, and (3) an available administrative health record at a community health center. In mainland China, community health centers establish administrative health records for local residents. These records contain comprehensive information, including details related to sociodemographic characteristics, children’s development, pregnancy and childbirth (for women), chronic disease diagnoses and management, and vaccination history. Chinese residents of a local area of more than six months are required to establish an administrative health record at their local community health center, regardless of age or permanent residency status. In this study, we anticipated that most parents who had resided in Shenzhen for over six months had already established such records.

A multistage random sampling method was employed to recruit participants. In the first stage, researchers entered the names of all community health centers in Shenzhen (926 in total) into an Excel spreadsheet, then used the “Random Cell Selection” function to randomly select 10 community health centers. In the second stage, researchers further randomly selected 200 residents with at least one child aged 0 to 15 from the administrative health records of each selected center. Details of the sampling method are reported in a published study [45]. Researchers contacted potential participants from the selected health centers by phone at various times during weekdays and weekends, introduced the study to them, and invited them to the community health center for face-to-face interviews. With written informed consent, the fieldworkers conducted interviews in Mandarin or Cantonese, which took about 30 min to complete. Upon completion of the interview, a cash coupon of CNY 25 (USD 3.5) was given to each participant as a token of appreciation. The institutional ethics committee of the Longhua District Center for Disease Control and Prevention approved this study (reference: 2024008).

### 2.3. Sample-Size Planning

The target sample size for the original cross-sectional survey was 1500. Assuming the level of behavioral intention to receive a seasonal influenza vaccination is 30% in the reference group (defined as participants who do not have a facilitating condition), the sample size is able to detect the smallest odds ratios of 1.36 between participants with and without a facilitating condition (power: 0.80; alpha: 0.05; PASS 11.0, NCSS, LLC, Kaysville, UT, USA).

### 2.4. Measurements

#### 2.4.1. Development of the Questionnaire

A panel consisting of experts in public health, vaccination behaviors, and health psychology; Centers for Disease Control and Prevention workers; and older adults was formed to develop the questionnaire. The procedures for questionnaire development and pilot testing were reported in a previously published study [45].

#### 2.4.2. Background Characteristics

Parents reported sociodemographic characteristics (e.g., age, sex assigned at birth, education level, marital status, and employment status), their history of confirmed SARS-CoV-2 infection and seasonal influenza vaccination uptake, and characteristics of their children (e.g., age, sex assigned at birth, number of siblings, and seasonal influenza vaccination uptake). Parents were asked to refer to the child whose birthday was closest to the survey date (the index child) when answering questions if they had at least two children aged 0–15 years within the household.

#### 2.4.3. Dependent Variable: Parents’ Intention to Receive Seasonal Influenza Vaccination

Participants were asked their likelihood of receiving seasonal influenza vaccination in the next year. The response categories were 1 = very unlikely, 2 = unlikely, 3 = neutral, 4 = likely, and 5 = very likely. Participants who answered “likely” or “very likely” were defined as having a behavioral intention to receive a seasonal influenza vaccination. The same methods to measure behavioral intention to receive vaccination were used in a previously published study [46].

#### 2.4.4. Independent Variables of Interest

Participants were also asked about their likelihood to vaccinate their children against seasonal influenza in the next year (response categories ranged from 1 = very unlikely to 5 = very likely). Participants who responded “likely” or “very likely” were defined as having a behavioral intention to vaccinate their children against seasonal influenza.

At the individual level, parents’ illness representation related to seasonal influenza was measured using the validated Chinese version of the Brief Illness Perception Questionnaire [47]. In this study, the term “disease” in the original tool was specified as “seasonal influenza”, and open-ended questions were excluded [48]. The B-IPQ includes eight items, each rated on a scale from 0 to 10, including (1) consequences (“How much does seasonal influenza affect your life?”), (2) timeline (“How long do you think your seasonal influenza will continue?”), (3) personal control (“How much control do you feel you have over your seasonal influenza?”), (4) treatment control (“How much do you think your treatment can help your seasonal influenza?”), (5) identity (“How much do you experience symptoms from your seasonal influenza?”), (6) concern (“How concerned are you about your seasonal influenza?”), (7) coherence (“How well do you feel you understand your seasonal influenza?”), and (8) emotions (“How much does your seasonal influenza affect you emotionally? e.g., does it make you angry, scared, upset or depressed?”) [49]. Higher scores for consequences, timeline, identity, concern, and emotions represent more negative illness perceptions, while higher scores for personal control, treatment control, and coherence indicate more positive perceptions.

Regarding independent variables at the interpersonal level, we used the validated Chinese version of the Elder Care Research Center Altruism Scale (Cronbach’s α of 0.88) to measure the level of general altruism [50]. Such a scale has been shown to be reliable to measure general altruism among both younger and older adults [51]. In addition, two questions measure family-oriented altruism—“Receiving seasonal influenza vaccination for yourself can protect your children” and “Receiving seasonal influenza vaccination for yourself can protect older adults or others in your household” (responses categories: 1 = disagree, 2 = neutral, 3 = agree)—with higher scores indicating a higher level of family-oriented altruism. Family harmony was assessed using the validated Chinese version of the 5-item Family Harmony Scale (Cronbach’s α > 0.80) [52], which includes five dimensions: effective communication, conflict resolution, mutual tolerance, family identity, and quality time spent with family. Each item was rated on a 5-point Likert scale, with responses ranging from 1 = strongly disagree to 5 = strongly agree. A higher score on the Family Harmony Scale indicates better family harmony. We adapted validated questions to assess the frequency of exposure to information related to seasonal influenza and other upper respiratory infections on common social media platforms in China (i.e., WeChat moments, WeChat group, Weibo, TikTok) in the past month [53]. These questions included exposure to information related to children’s symptoms after upper respiratory infection shared by parents, increases in hospital admission due to influenza and other upper respiratory infections, and the number of new cases and severe cases of influenza and other upper respiratory infections. The response categories were 1 = almost none, 2 = seldom, 3 = sometimes, and 4 = always. Higher scores indicate a higher frequency of information exposure. In addition, one validated item was used to measure the frequency of thoughtful consideration of the veracity of vaccine-specific information on social media platforms.

### 2.5. Statistical Analysis

We present frequencies for categorical variables and medians with interquartile ranges (IQRs) for continuous variables. The Cronbach’ alpha of the scale was calculated using reliability tests. Parents’ intention to receive a seasonal influenza vaccination is the dependent variable. We followed the statistical analysis methods reported in numerous published studies [54]. Univariate logistic regression models were first used to investigate the associations between each background characteristic and the dependent variable. Background characteristics with *p* < 0.05 in univariate analyses were subsequently adjusted in the multivariate logistic regression models. Each multivariate logistic regression model contained one independent variable of interest and all significant background characteristics. Multicollinearity in each model was assessed using variance inflation factors, with values below 5 indicating no significant multicollinearity. Subgroup analyses were conducted among parents who were 24–40 years old and over 40 years old. Crude odds ratios (ORs), adjusted odds ratios (AORs), and their 95% confidence interval (CI) were obtained. All statistical analyses mentioned above were performed via R version 4.2.3. Two-sided *p* values < 0.05 were considered significant.

## 3. Results

### 3.1. Background Characteristics

Among the 2000 parents approached, 131 had migrated to other cities at the time of this study, 365 refused to participate in the study due to a lack of time or other tangible reasons, and 1504 completed the survey. In this study, the majority of surveyed parents were aged 31–40 (60.5%), were female (65.7%), held tertiary or above education (77.2%), had an income of over CNY 5000 (USD 701 USD) (75.6%), were permanent Shenzhen residents (63.4%), and were married (97.1%). Most of them reported a history of confirmed SARS-CoV-2 infection (81.8%) and the receipt of COVID-19 vaccination booster doses (74.1%). Between September 2023 and August 2024, 9.8% and 15.1% of the parents reported a history of confirmed seasonal influenza infection and seasonal influenza vaccination, respectively. Among their index children, over half were aged 0–6 years (59.1%), male (52.4%), and had at least one sibling (59.4%); 36.4% of the index children received a seasonal influenza vaccination during the 2023/24 flu season (Table 1).

### 3.2. Behavioral Intention to Receive Seasonal Influenza Vaccination and Descriptive Statistics of Independent Variables of Interest

Among the parents, 47.6% intended to receive a seasonal influenza vaccination for themselves in the next year, and 57.5% of them intended to vaccinate their children against seasonal influenza in the next year. The median scores and IQR of the item related to illness representation of seasonal influenza are presented in Table 2. For interpersonal-level variables, the median scores were 18.0 (17.0, 20.0) for the Elder Care Research Center Altruism Scale and 20.0 (20.0, 21.0) for the Family Harmony Scale. Most parents agreed that receiving seasonal influenza vaccination for themselves could protect their children (83.8%) and older adults or others in their household (85.0%). Regarding information exposure, around 50% of participants were sometimes/always exposed to information related to children’s symptoms after upper respiratory infection shared by parents (49.1%), increases in hospital admission due to influenza and other upper respiratory infections (58.9%), and the number of new cases and severe cases of influenza and other upper respiratory infections (51.6%). Over half of the parents sometimes/always considered the veracity of vaccine-specific information on social media platforms (55.6%) (Table 2).

### 3.3. Associations Between Background Characteristics and Parents’ Intention to Receive a Seasonal Influenza Vaccination

In the univariate analysis, parents who were older (41–50 years: OR: 0.66, 95% CI: 0.45, 0.98, *p* = 0.04; ≥50 years: OR: 0.57, 95% CI: 0.33, 0.96, *p* = 0.04; reference group: 31–40 years), without full-time employment (OR: 0.73, 95% CI: 0.59, 0.91, *p* = 0.01), and with more than one child (2 children: OR: 0.76, 95% CI: 0.61, 0.94, *p* = 0.01; ≥3 children: OR: 0.61, 95% CI: 0.41, 0.92, *p* = 0.02; reference group: 1 child) had a lower likelihood of intending to receive a seasonal influenza vaccination in the next year. History of seasonal influenza vaccination among the parents (OR: 2.62, 95% CI: 1.95, 3.54, *p* < 0.001), history of seasonal influenza vaccination of the index children in the 2023/24 flu season (OR: 1.47, 95% CI: 1.19, 1,81, *p* < 0.001), and the index child having received two doses of COVID-19 vaccination (OR: 1.31, 95% CI: 1.02, 1.68, *p* = 0.03; reference group: 0–1 dose) were associated with a higher likelihood of intending to receive a seasonal influenza vaccination among the parents (Table 1).

### 3.4. Factors Associated with Parents’ Intention to Receive a Seasonal Influenza Vaccination

After adjusting for these significant background characteristics, parents with intention to receive a seasonal influenza vaccination for themselves had a higher likelihood of intending to vaccinate their children against seasonal influenza in the next year (AOR: 20.39, 95% CI: 15.26, 27.25, *p* < 0.001). Regarding independent variables of interest at the individual level, parents who perceived more negative effects of seasonal influenza on their lives (consequences) (AOR: 1.25, 95% CI: 1.20, 1.31, *p* < 0.001), longer duration of seasonal influenza infection (timeline) (AOR: 1.23, 95% CI: 1.17, 1.29, *p* < 0.001), that seasonal influenza infection could be controlled by themselves (personal control) (AOR: 1.15, 95% CI: 1.10, 1.21, *p* < 0.001) or medical treatment (treatment control) (AOR: 1.25, 95% CI: 1.19, 1.30, *p* < 0.001), and severer symptoms caused by seasonal influenza infection (identity) (AOR: 1.21, 95% CI: 1.15, 1.27, *p* < 0.001), as well as those with more concerns (concern) (AOR: 1.20, 95% CI: 1.14, 1.26, *p* < 0.001) and a better understanding of seasonal influenza (coherence) (AOR: 1.23, 95% CI: 1.17, 1.29, *p* < 0.001) and with more negative emotional responses to seasonal influenza (emotions) (AOR: 1.17, 95% CI: 1.12, 1.23, *p* < 0.001) were more likely to have a behavioral intention to receive a seasonal influenza vaccination (Table 3).

Regarding variables at the interpersonal level, a higher level of general altruism (AOR: 1.10, 95% CI: 1.07, 1.14, *p* < 0.001), beliefs that receiving seasonal influenza vaccination for themselves could protect their children (AOR: 2.39, 95% CI: 1.89, 3.04, *p* < 0.001) or other household members (AOR: 2.47, 95% CI: 1.93, 3.17, *p* < 0.001), and better family harmony (AOR: 1.07, 95% CI: 1.04, 1.10, *p* < 0.001) were associated with higher odds of parents having a behavioral intention to receive a seasonal influenza vaccination. In addition, parents who had a higher frequency of exposure to information related to children’s symptoms after upper respiratory infection shared by parents (AOR: 1.24, 95% CI: 1.12, 1.39, *p* < 0.001), increases in hospital admission due to influenza and other upper respiratory infections (AOR: 1.38, 95% CI: 1.24, 1.53, *p* < 0.001), and the number of new cases and severe cases of influenza and other upper respiratory infections (AOR: 1.31, 95% CI: 1.18, 1.46, *p* < 0.001) were more likely to have a behavioral intention to receive a seasonal influenza vaccination. Moreover, a higher frequency of thoughtful consideration of the veracity of vaccine-specific information on social media was associated with a higher likelihood of intending to receive a seasonal influenza vaccination among parents (AOR: 1.33, 95% CI: 1.20, 1.47, *p* < 0.001) (Table 3). No significant multicollinearity was detected across the models, with all variance inflation factor values below 5.

### 3.5. Subgroup Analysis

Similar factors were associated with behavioral intention to receive a seasonal influenza vaccination among parents of different age groups, with the exception of two variables on information exposure. The frequency of exposure to information related to children’s symptoms after upper respiratory infection shared by parents and the number of new cases and severe cases of influenza and other upper respiratory infection were significantly associated with behavioral intention to receive a seasonal influenza vaccination among parents aged over 40 years. However, the associations between these two variables and the dependent variable were statistically non-significant among parents aged 24–40 years (Table A1).

## 4. Discussion

To the best of our knowledge, this is one of the first studies to investigate the influences of illness representation, altruism, family harmony, and exposure to information through social media platforms on parents’ intention to receive a seasonal influenza vaccination to protect themselves. This study expanded the application of the Socio-ecological Model. Moreover, our findings may have practical implications to guide the development of health promotion in the future. Other strengths of this study include a relatively large sample size and the use of multistage random sampling to recruit the participants.

In this study, 47.6% of parents intended to receive a seasonal influenza vaccination to protect themselves. Such a level of behavioral intention is similar to that reported among parents in other Chinese cities (42.6–53.8%) and Spain (49.2%) [21,55,56]. However, as compared to older adults (82.1–86.0%) [57,58] and healthcare workers in China (74.9–79.6%) [59], a lower proportion of parents in our study reported an intention to receive a seasonal influenza vaccination. Most parents in this study were young. They might perceive a lower threat of seasonal influenza compared to older adults. Hence, there is a strong need and considerable opportunity to promote seasonal influenza vaccination among younger adults in China.

Similar to the findings of previous studies, parents who were older and without full-time employment were less likely to report an intention to receive a seasonal influenza vaccination for themselves [21,22]. As compared to their younger counterparts, older parents perceived seasonal influenza vaccination as less effective for themselves [58,60]. Some employers in China encourage their employees to receive a seasonal influenza vaccination through various strategies, such as the dissemination health communication messages and providing on-site vaccination, which may increase employees’ motivation to receive a seasonal influenza vaccination [61]. Having more children was associated with lower odds of intending to receive a seasonal influenza vaccination. Similar findings were reported by previous studies [22]. Parents with more children might have higher childcare burdens and less time to receive a seasonal influenza vaccination. Therefore, in future seasonal influenza vaccination promotion campaigns, more attention should be paid to parents who are older, without full-time employment, and with more children. Seasonal influenza vaccination uptake among both parents and their children, as well as COVID-19 vaccination uptake among their children, was associated with a higher likelihood of intending to receive a seasonal influenza vaccination among parents. Parents with such vaccination history might have more motivation to use vaccination to prevent infectious diseases.

Our findings provide have empirical implications for the promotion of seasonal influenza vaccination. In line with previous studies, parents with an intention to receive a seasonal influenza vaccination for themselves were more likely to vaccinate their children against seasonal influenza [56,62,63,64]. Therefore, promoting seasonal influenza vaccination among parents could increase seasonal influenza vaccination coverage among two groups with the lowest uptake rate in China: adults aged 18–59 years and children aged under 18 years [10,11]. At the individual level, information about seasonal influenza disease perceptions could be incorporated into health promotion campaigns to promote seasonal influenza vaccination. Parents who perceived more negative consequences of seasonal influenza in their lives (consequence), longer timeline of seasonal influenza infection (timeline), and more severe symptoms caused by seasonal influenza infection (identity) were more likely to report an intention to receive a seasonal influenza vaccination. Therefore, it may be helpful to emphasize the efficacy of seasonal influenza vaccination in preventing severe and long-term consequences of seasonal influenza in health communication messages. Higher perceived personal and treatment control of seasonal influenza was associated with a higher intention to receive a seasonal influenza vaccination. Individuals who perceive higher personal or treatment control may exhibit stronger confidence in vaccine efficacy, a facilitator of vaccination uptake [48]. Parents with a better understanding of seasonal influenza (coherence) were more likely to report an intention to receive a seasonal influenza vaccination, consistent with findings from another study of parents [21]. Comprehensive health education for parents can impove knowledge of seasonal influenza, thereby promoting seasonal influenza vaccination uptake. Parents who express greater concern and more negative emotions about influenza are more likely to receive a seasonal influenza vaccination, as found in other vaccination studies [29,48]. Future initiatives should consider encouraging parents with concerns and negative emotions to receive seasonal influenza vaccination, transforming these negative emotions into preventive behavior.

At the interpersonal level, parents with higher levels of altruism and family harmony were more likely to report an intention to receive seasonal influenza vaccination. Similar to previous studies [51,65], parents with higher levels of general altruism were more likely to have an intention to receive seasonal influenza vaccination. Individuals with a stronger desire to protect others may place greater emphasis on the societal health benefits of vaccination, thereby choosing to get vaccinated [66]. Family-oriented altruism was associated with a higher likelihood of intending to receive a seasonal influenza vaccination. Parents with better family harmony may receive stronger social support from their families, which can help them develop healthy behaviors (e.g., vaccination) [67]. Future health promotion should emphasize herd immunity through seasonal influenza vaccination, particularly the role of families in health decision making [68]. Seasonal influenza appears to be a popular topic on social media, as over 50% of parents were always/sometimes exposed to information related to seasonal influenza in the past month on social media. In line with previous studies [48,62], higher levels of exposure to information related to seasonal influenza vaccination (e.g., statistics on seasonal influenza and other upper respiratory infections and parents’ sharing of children’s symptoms) on social media were associated with higher intentions among parents to receive the seasonal influenza vaccination. Health authorities should consider the use of social media to deliver health promotion messages related to seasonal influenza vaccination, particularly on official social media platforms, which are perceived as reliable sources of information [69]. We found that the proportion of parents who always thought carefully about the veracity of information specific to seasonal influenza vaccination remains low, at only about 20%. However, thoughtful consideration of the veracity of information specific to seasonal influenza vaccination plays an important role in promoting vaccination uptake, as confirmed by other studies [48,62,70]. Health authorities should proactively identify misinformation related to seasonal influenza vaccination and promptly clarify it through official communication channels. At the same time, efforts should be made to enhance parents’ health literacy and strengthen their ability to critically evaluate health information concerning themselves and their children.

Subgroup analysis revealed that parents aged over 40 years were more sensitive to information about children’s symptoms shared by other parents and the number of new cases and severe cases of upper respiratory infection on social media than their younger counterparts. As compared to younger people, older adults often have higher risk perceptions for influenza, and vaccine information on social media, particularly content centered on risks, may further enforce this risk perception, thereby increasing their vaccination intentions [71,72].

This study has several limitations. First, we did not collect information from parents who declined to participate in the study. It is possible that participants and those who refused to participate have different characteristics. Self-selection bias existed. Second, since the interviews were conducted in community health centers, taking up a vaccine and intending to receive a seasonal influenza vaccination might be considered socially desirable by the participants. Parents might have over-reported their behavioral intention to receive a seasonal influenza vaccination. Third, due to differences in healthcare systems, vaccination delivery models, health literacy, and cultural factors, the findings might not be applicable to other parts of China. Shenzhen is one of the most developed cities in mainland China. The performance of its healthcare system and residents’ health literacy might be higher than in less developed regions of the country. Therefore, it is expected that the level of behavioral intention to receive a seasonal influenza vaccination would be higher among Shenzhen residents compared to those living in less developed regions. Fourth, we did not include questions related to economic advantages of seasonal influenza vaccination compared with treatment of seasonal influenza. Perceived economic advantages of seasonal influenza vaccination might be a facilitator of vaccination uptake. Moreover, this study did not measure the presence of comorbidities. The presence of comorbidities is associated with higher levels of motivation to receive a seasonal influenza vaccination, especially among older adults [73]. However, most of our participants were 24–40 years old. It is expected the prevalence of chronic conditions among younger adults is much lower than that among older adults in China [74]. Furthermore, many younger adults in China are unaware of their risk of chronic conditions [75]. Therefore, the impact of comorbidities on intention to receive a seasonal influenza vaccination might be limited in our sample. Last but not the least, this was a cross-sectional survey and could not establish causal relationships.

## 5. Conclusions

In summary, relatively few parents with a child aged 0–15 years reported a behavioral intention to receive a seasonal influenza vaccination in the next year. More efforts are needed to promote seasonal influenza vaccination in this group, among parents who are older, without full-time employment, and with more children. Modifying disease perceptions related to seasonal influenza and emphasizing herd immunity through seasonal influenza vaccination might be useful in increasing seasonal influenza vaccination uptake. Health authorities should consider using social media as a communication channel to promote seasonal influenza vaccination.

## Figures and Tables

**Table 1:** Background characteristics of the parents and their index children and their associations with parents’ intention to receive seasonal influenza vaccination in the next year (n = 1504).

	n (%)	OR (95% CI)	*p* Values
Background characteristics of the parents			
Age group, years			
24–30	145 (9.6)	1.00	
31–40	910 (60.5)	0.86 (0.61, 1.23)	0.41
41–50	357 (23.7)	0.66 (0.45, 0.98)	0.04
≥50	92 (6.1)	0.57 (0.33, 0.96)	0.04
Sex assigned at birth			
Male	516 (34.3)	1.00	
Female	988 (65.7)	1.22 (0.98, 1.51)	0.07
Education level			
Secondary or below	343 (22.8)	1.00	
Tertiary or above	1161 (77.2)	1.21 (0.95, 1.54)	0.13
Current relationship status			
Married	1460 (97.1)	1.00	
Single/divorced/separated/widowed	44 (2.9)	1.33 (0.73, 2.43)	0.35
Current employment status			
Full-time	1018 (67.7)	1.00	
Part-time/self-employed/unemployed/housewife	486 (32.3)	0.73 (0.59, 0.91)	0.01
Monthly personal income level			
≤CNY 4999 (USD 689)	366 (24.3)	1.00	
CNY 5000–9999 (USD 690–1379)	572 (38.0)	1.27 (0.98, 1.66)	0.07
≥CNY 10,000 (USD 1379)	566 (37.6)	1.09 (0.84, 1.42)	0.53
Permanent residents of Shenzhen			
Yes	953 (63.4)	1.00	
No	551 (36.6)	0.91 (0.74, 1.12)	0.37
History of confirmed SARS-CoV-2 infection			
No	273 (18.2)	1.00	
Yes	1231 (81.8)	1.09 (0.84, 1.42)	0.51
History of confirmed seasonal influenza infection in the 2023/24 flu season (September 2023 to August 2024)			
No/uncertain	1358 (90.3)	1.00	
Yes	146 (9.7)	0.90 (0.64, 1.27)	0.54
Number of doses of COVID-19 vaccination			
0–1	103 (6.9)	1.00	
2	286 (19.0)	0.92 (0.58, 1.46)	0.74
≥3	1115 (74.1)	1.40 (0.93, 2.11)	0.10
Seasonal influenza vaccination uptake for the 2023/24 flu season (September 2023 to August 2024)			
No/uncertain	1277 (84.9)	1.00	
Yes	227 (15.1)	2.62 (1.95, 3.54)	<0.001
Background characteristics of the index child			
Age group, years			
0–3	328 (21.8)	1.00	
4–6	561 (37.3)	1.31 (0.99, 1.72)	0.06
7–10	512 (24.0)	0.93 (0.70, 1.23)	0.61
≥10	103 (6.9)	1.00 (0.64, 1.55)	0.99
Sex assigned at birth			
Male	788 (52.4)	1.00	
Female	716 (47.6)	0.82 (0.67, 1.00)	0.051
Number of siblings			
0	612 (40.7)	1.00	
1	777 (51.7)	0.76 (0.61, 0.94)	0.01
≥2	115 (7.7)	0.61 (0.41, 0.92)	0.02
Number of doses of COVID-19 vaccination			
0–1	505 (33.6)	1.00	
2	501 (33.3)	1.31 (1.02, 1.68)	0.03
≥3	498 (33.1)	1.13 (0.88, 1.45)	0.34
Seasonal influenza vaccination uptake for the 2023/24 flu season (September 2023 to August 2024)			
No/uncertain	956 (63.6)	1.00	
Yes	548 (36.4)	1.47 (1.19, 1.81)	<0.001

OR: crude odds ratio obtained from univariate logistic regression models. CI: confidence interval.

**Table 2:** Parents’ intention to receive a seasonal influenza vaccination in the next year and descriptive statistics of independent variables of interest.

	n (%)	Median (IQR)
Parents’ intention to receive seasonal influenza vaccination		
Likelihood of receiving seasonal influenza vaccination in the next year		
Very unlikely/unlikely/neutral	788 (52.4)	N.A.
Likely/very likely	716 (47.6)	N.A.
Intention to vaccinate their children against seasonal influenza		
Likelihood of vaccinating their children against seasonal influenza in the next year		
Very unlikely/unlikely/neutral	639 (42.5)	N.A.
Likely/very likely	865 (57.5)	N.A.
Individual-level variables		
Brief Illness Perception Questionnaire related to seasonal influenza		
Domains of the BIPQ		
Consequences	N.A.	5.0 (4.0, 7.0)
Timeline	N.A.	5.0 (4.0, 7.0)
Personal control	N.A.	5.0 (4.0, 7.0)
Treatment control	N.A.	7.0 (5.0, 8.0)
Identity	N.A.	6.0 (5.0, 7.5)
Concern	N.A.	7.0 (5.0, 8.0)
Coherence	N.A.	6.0 (5.0, 7.0)
Emotions	N.A.	5.0 (4.0, 7.0)
Interpersonal-level variables		
Altruistic factors		
Elder Care Research Center Altruism Scale		
Scale score	N.A.	18.0 (17.0, 20.0)
Receiving seasonal influenza vaccination for yourself can protect your children, agree	1260 (83.8)	N.A.
Item score	N.A.	3.0 (3.0, 3.0)
Receiving seasonal influenza vaccination for yourself can protect older adults or others in your household, agree	1279 (85.0)	N.A.
Item score	N.A.	3.0 (3.0, 3.0)
Family Harmony		
Family Harmony Scale		
Scale score	N.A.	20.0 (20.0, 21.0)
Information exposure		
Frequency of exposure to the following information on social media in the past month		
Children’s symptoms after upper respiratory infection shared by parents		
Almost none	347 (23.1)	N.A.
Seldom	418 (27.8)	N.A.
Sometimes	543 (36.1)	N.A.
Always	196 (13.0)	N.A.
Item score	N.A.	2.0 (2.0, 3.0)
Increase in hospital admission due to influenza and other upper respiratory infections		
Almost none	288 (19.1)	N.A.
Seldom	330 (21.9)	N.A.
Sometimes	537 (35.7)	N.A.
Always	349 (23.2)	N.A.
Item score	N.A.	3.0 (2.0, 3.0)
Number of new cases and severe cases of influenza and other upper respiratory infections		
Almost none	324 (21.5)	N.A.
Seldom	404 (26.9)	N.A.
Sometimes	551 (36.6)	N.A.
Always	225 (15.0)	N.A.
Item score	N.A.	3.0 (2.0, 3.0)
Thoughtful consideration of the veracity of vaccine-specific information		
Almost none	320 (21.3)	N.A.
Seldom	347 (23.1)	N.A.
Sometimes	525 (34.9)	N.A.
Always	312 (20.7)	N.A.
Item score	N.A.	3.0 (2.0, 3.0)

N.A.: not applicable.

**Table 3:** Factors associated with parental intention to receive seasonal influenza vaccination in the next year.

	AOR (95% CI) ^1^	*p* Value
Likelihood to vaccinate their children against seasonal influenza in the next year (Likely/very likely)	20.39 (15.26, 27.25)	<0.001
Individual-level variables		
Domains of the BIPQ (Score)		
Consequences	1.25 (1.20, 1.31)	<0.001
Timeline	1.23 (1.17, 1.29)	<0.001
Personal control	1.15 (1.10, 1.21)	<0.001
Treatment control	1.25 (1.19, 1.30)	<0.001
Identity	1.21 (1.15, 1.27)	<0.001
Concern	1.20 (1.14, 1.26)	<0.001
Coherence	1.23 (1.17, 1.29)	<0.001
Emotions	1.17 (1.12, 1.23)	<0.001
Interpersonal-level variables		
Elder Care Research Center Altruism Scale		
Scale score	1.10 (1.07, 1.14)	<0.001
Receiving seasonal influenza vaccination for yourself can protect children		
Item score	2.39 (1.89, 3.04)	<0.001
Receiving seasonal influenza vaccination for yourself can protect older adults or others in the household		
Item score	2.47 (1.93, 3.17)	<0.001
Family Harmony Scale		
Scale score	1.07 (1.04, 1.10)	<0.001
Frequency of exposure to the following information on social media in the past month		
Children’s symptoms after upper respiratory infection shared by parents		
Item score	1.24 (1.12, 1.39)	<0.001
Increase in hospital admission due to influenza and other upper respiratory infections		
Item score	1.38 (1.24, 1.53)	<0.001
Number of new cases and severe cases of influenza and other upper respiratory infections		
Item score	1.31 (1.18, 1.46)	<0.001
Thoughtful consideration of the veracity of vaccine-specific information		
Item score	1.33 (1.20, 1.47)	<0.001

^1^ AOR: adjusted odds ratio obtained by multivariable logistic regression models, adjusted for significant background characteristics listed in Table 2.

## Data Availability

The datasets generated and/or analyzed during the current study are not publicly available, as they contain sensitive personal information, but they are available from the corresponding author upon reasonable request.

## Appendix A

**Table A1:** Subgroup analysis of factors associated with parental intention to receive seasonal influenza vaccination in the next year by age group.

	Age Group			
	24–40 Years		>40 Years	
	AOR (95% CI) ^1^	*p* Values	AOR (95% CI) ^1^	*p* Values
Likelihood of vaccinating their children against seasonal influenza in the next year (Likely/very likely)	18.82 (11.79, 30.05)	<0.001	22.81 (10.45, 49.75)	<0.001
Individual level variables				
Domains of the BIPQ (Score)				
Consequences	1.28 (1.18, 1.38)	<0.001	1.26 (1.12, 1.41)	<0.001
Timeline	1.28 (1.18, 1.39)	<0.001	1.22 (1.08, 1.38)	0.001
Personal control	1.18 (1.09, 1.28)	<0.001	1.14 (1.01, 1.28)	0.03
Treatment control	1.29 (1.19, 1.40)	<0.001	1.21 (1.07, 1.36)	0.002
Identity	1.24 (1.14, 1.35)	<0.001	1.19 (1.05, 1.34)	0.005
Concern	1.32 (1.22, 1.44)	<0.001	1.22 (1.08, 1.36)	0.001
Coherence	1.31 (1.20, 1.43)	<0.001	1.22 (1.08, 1.38)	0.002
Emotions	1.24 (1.14, 1.34)	<0.001	1.21 (1.07, 1.36)	0.002
Interpersonal-level variables				
Elder Care Research Center Altruism Scale				
Scale score	1.12 (1.06, 1.19)	<0.001	1.18 (1.07, 1.30)	0.001
Receiving seasonal influenza vaccination for yourself can protect children				
Item score	2.45 (1.68, 3.58)	<0.001	4.68 (2.18, 10.04)	<0.001
Receiving seasonal influenza vaccination for yourself can protect older adults or others in the household				
Item score	2.62 (1.76, 3.91)	<0.001	4.67 (2.09, 10.46)	<0.001
Family Harmony Scale				
Scale score	1.06 (1.01, 1.10)	0.01	1.16 (1.07, 1.26)	<0.001
Frequency of exposure to the following information on social media in the past month				
Children’s symptoms after upper respiratory infection shared by parents				
Item score	1.19 (1.00, 1.42)	0.056	1.42 (1.05, 1.92)	0.03
Increase in hospital admission due to influenza and other upper respiratory infections				
Item score	1.26 (1.06, 1.50)	0.008	1.73 (1.28, 2.35)	<0.001
Number of new cases and severe cases of influenza and other upper respiratory infections				
Item score	1.12 (0.94, 1.33)	0.21	1.68 (1.23, 2.30)	0.001
Thoughtful consideration of the veracity of vaccine-specific information				
Item score	1.19 (1.01, 1.41)	0.04	1.83 (1.36, 2.46)	<0.001

^1^ AOR: adjusted odds ratio obtained by multivariable logistic regression models, adjusted for significant background characteristics listed in Table 2, except for age.
